# Echocardiographic Probability of Pulmonary Hypertension, Cardiac Structural Alterations and All‐Cause Mortality in Maintenance Hemodialysis Patients: A Single‐Center Retrospective Cohort Study With Competing Risk Analysis

**DOI:** 10.1002/clc.70415

**Published:** 2026-07-18

**Authors:** Jingwen Zhou, Zhenhua Yang, Dongmei Huo

**Affiliations:** ^1^ Department of Nephrology the First Affiliated Hospital of Guangxi Medical University Nanning Guangxi Zhuang Autonomous Region China

**Keywords:** all‐cause mortality, competing risk model, echocardiographic probability of pulmonary hypertension, maintenance hemodialysis, right ventricular remodeling, vascular access

## Abstract

**Background:**

Maintenance hemodialysis (MHD) patients carry extremely high cardiovascular mortality, where pulmonary hypertension (PH) and vascular access are critical prognostic factors. However, previous survival studies seldom considered renal transplantation as a competing event, potentially biasing risk factor estimates, and the heterogeneity of mortality predictors across vascular access subtypes remains unclear. This study aimed to evaluate whether echocardiographic probability of PH is independently associated with all‐cause mortality in MHD patients using a competing risk model. Secondary objectives included identifying additional clinical and echocardiographic predictors of mortality and comparing survival between vascular access types. Exploratory subgroup analyses by vascular access and construction of a nomogram were also performed.

**Methods:**

This single‐center retrospective cohort enrolled 749 MHD patients from the First Affiliated Hospital of Guangxi Medical University between May 2010 and May 2022. All‐cause mortality was the primary endpoint, with renal transplantation as the competing event. Independent predictors were identified via the Fine‐Gray model, and subgroup analyses were performed by tunneled cuffed catheter (TCC) and arteriovenous fistula/graft (AVF/AVG).

**Results:**

During follow‐up, 274 deaths and 64 renal transplantations occurred. Intermediate/high probability of PH, age, left atrial diameter, and right ventricular diameter (RVD) independently predicted mortality. Subgroups showed age and high PH predicted death in TCC patients, whereas age, intermediate/high PH, and RVD predicted death in AVF/AVG patients. No survival difference was found between groups.

**Conclusion:**

PH severity, age, and cardiac structural remodeling are independent mortality risk factors in MHD patients. Risk predictors vary by vascular access, and individualized cardiovascular stratification may improve long‐term outcomes.

## Graphical abstract

Using a Fine‑Gray competing risk model accounting for renal transplantation, pulmonary hypertension probability, age, and right ventricular diameter independently predicted mortality in maintenance hemodialysis patients. Risk profiles differed by vascular access, supporting individualized cardiovascular risk stratification.

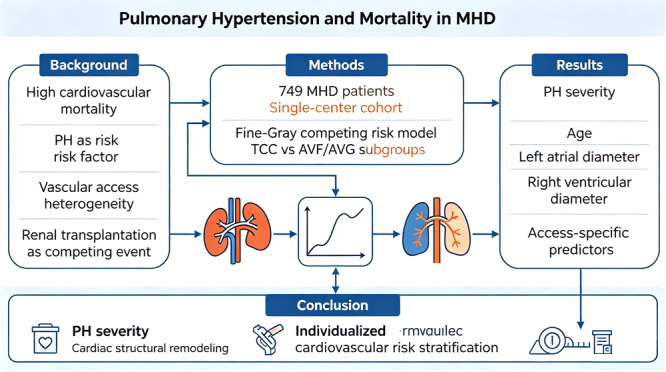

## 1 Introduction

Chronic kidney disease (CKD) represents a growing global public health burden, with an estimated global prevalence of 14.3% [1]. In China alone, CKD affects approximately 132.3 million individuals, accounting for one‐sixth of the total population [2]. A cross‐sectional epidemiological study in China reported that 1.34% of patients with CKD progress to end‐stage kidney disease (ESRD), a population predominantly composed of older adults, with 18.3% of ESRD patients suffering from CKD‐related cardiovascular complications [3]. Given the substantial global healthcare burden of CKD management, renal replacement therapy (RRT), including hemodialysis (HD), renal transplantation, and peritoneal dialysis, is a life‐sustaining necessity for patients with ESKD [4].

In the United States, approximately 400,000 patients are currently receiving maintenance hemodialysis (MHD) [5]. Despite continuous improvements in dialysis quality and care standards, MHD patients still face excessively high hospitalization rates, impaired quality of life, and elevated mortality. The annual all‐cause mortality rate in this population remains above 20%, which is 10 times higher than that in the age‐matched general population [6]. Regardless of dialysis modality, the 5‐year survival rate of MHD patients is only around 40%, which is inferior to the prognosis of most malignant tumors [6, 7]. In China, more than 90% of patients with uremia receive HD treatment, which effectively prolongs survival and reduces the risk of premature death [2]. Nevertheless, the cardiovascular disease (CVD)‐related mortality rate of dialysis patients is 6.5–7.9 times higher than that of the general population [8], and CVD remains the leading cause of death in HD patients, with more than half of all fatal events attributed to CVD. Cardiovascular events (CVEs), mainly driven by hypertension and left ventricular hypertrophy (LVH), are the primary contributors to increased mortality in the HD population. Chronic subclinical volume overload is highly prevalent—nearly universal in HD patients receiving standard thrice‐weekly treatment regimens—and is directly associated with hypertension, accelerated arteriosclerosis, LVH, heart failure, and ultimately increased morbidity and mortality.

CVD‐related deaths account for 40% of all‐cause mortality in MHD patients, with the majority of fatalities resulting from heart failure, acute myocardial infarction, and life‐threatening arrhythmias [9, 10]. The profile of cardiovascular dysfunction observed in dialysis patients is distinct from that in the general population. Although traditional cardiovascular risk factors are highly prevalent in patients with ESKD, they only partially explain the excessive cardiovascular morbidity and mortality in this population. Previous studies have predominantly focused on left ventricular structural and functional alterations in HD patients, with relatively limited attention to the impact on the right‐sided heart. However, with the deepening understanding of disease pathophysiology, the creation of an arteriovenous fistula (AVF) for hemodialysis access induces significant hemodynamic shunting and has been recognized to contribute to both left‐ and right‐ventricular systolic dysfunction, which play a critical role in the survival of HD patients [11, 12]. Notably, the impact of right ventricular structural alterations on clinical outcomes remains understudied. Changes in right ventricular morphology have been confirmed to be significantly associated with the phenotype of pulmonary hypertension (PH), hemodynamic status, and right ventricular function [13, 14, 15]. To date, large‐scale data delineating the differential impact of right ventricular structural changes on long‐term survival in MHD patients remain scarce. Meanwhile, renal transplantation, as the optimal RRT modality for patients with kidney failure, has been increasingly valued and widely applied worldwide, as it significantly improves patient prognosis and long‐term survival.

Against this background, the primary objective of this retrospective single‐center cohort study was to determine whether echocardiographically defined PH probability is independently associated with all‐cause mortality in MHD patients, accounting for renal transplantation as a competing event. Secondary objectives included identifying other clinical and echocardiographic predictors of mortality and comparing survival between TCC and AVF/AVG groups. Exploratory analyses examined heterogeneity in risk factors across vascular access subtypes and developed a nomogram for individualized risk prediction.

## 2 Materials and Methods

### 2.1 Study Design and Study Population

This single‐center retrospective cohort study was conducted on patients receiving MHD at the First Affiliated Hospital of Guangxi Medical University between May 2010 and May 2022. The study protocol was in accordance with the ethical guidelines of the Declaration of Helsinki. Ethical approval was obtained from the Institutional Review Board of the First Affiliated Hospital of Guangxi Medical University, and the requirement for written informed consent was waived due to the retrospective, non‐interventional nature of the study.

#### 2.1.1 Inclusion Criteria

1. Patients aged between 18 and 85 years old;
2. Patients with ESKD who received MHD for at least 3 months, with a hemodialysis regimen of three sessions per week, 4 h per session, in accordance with the 2012 Kidney Disease: Improving Global Outcomes (KDIGO) Clinical Practice Guidelines and the Chinese Clinical Practice Guideline for Hemodialysis Adequacy. Eligible patients had an interdialytic weight gain of no more than 5% of dry body weight, a urea reduction ratio (URR) > 60%, and a single‐pool Kt/V (spKt/V) of 1.2–1.4 [16];
3. All patients received hemodialysis with a blood flow rate of 250–300 mL/min, a dialysate flow rate of 500 mL/min, and bicarbonate‐buffered dialysate. The ultrafiltration volume was determined based on the patient's dry body weight, which was defined as the weight at which no clinical signs of edema or volume overload were present, no intradialytic hypotension, muscle spasm, nausea, or vomiting occurred during the second half of the dialysis session, and was reassessed regularly for each patient.

#### 2.1.2 Exclusion Criteria

1. Patients aged < 18 years or > 85 years;
2. Pregnant women, or patients with a history of heart failure, congenital heart disease, pulmonary embolism, or connective tissue disease;
3. Patients using two types of hemodialysis vascular access simultaneously;
4. Patients receiving concurrent peritoneal dialysis with hemodialysis;
5. Patients with severe inflammatory conditions, including severe pneumonia, septic shock, or sepsis;
6. Patients with a history of major central vascular surgery;
7. Patients with incomplete medical records or unavailable clinical and follow‐up data.

Participant flow and selection. A total of 757 patients receiving maintenance hemodialysis at our center between May 2010 and May 2022 were initially screened. After applying the inclusion and exclusion criteria, 749 patients were eligible for the competing risk analysis. Nine patients were excluded due to missing outcome data (loss to follow‐up without documented death or transplantation). The final analytic cohort comprised 749 patients, of whom 685 had complete baseline echocardiographic and laboratory data.

### 2.2 Data Collection

Baseline data were defined as clinical and laboratory parameters collected within 1 week before and after the first TTE examination after MHD initiation. The primary endpoint of the study was all‐cause mortality, and we aimed to identify the risk factors for all‐cause mortality in MHD patients, and further explore the independent predictors of survival in the presence of competing risk events using competing risk regression models.

The following data were collected for each eligible patient:

1. Baseline demographic and clinical characteristics: including sex, age, vintage of hemodialysis, height, body weight, blood pressure, pulse rate, and comorbidities at baseline.
2. Laboratory parameters: including routine blood test, serum creatinine (sCr), blood urea nitrogen (BUN), fasting blood glucose (GLU), serum albumin (ALB), prealbumin, total cholesterol (TC), triglycerides (TG), intact parathyroid hormone (iPTH), C‐reactive protein (CRP), serum iron, serum ferritin, serum calcium, potassium, chloride, magnesium, phosphorus, prothrombin time, and fibrinogen.
3. Clinical and follow‐up data: including type of hemodialysis vascular access, age at hemodialysis initiation, survival time, date of hemodialysis initiation, interdialytic ultrafiltration volume, and final clinical outcome. Survival status was obtained through inpatient and outpatient medical records, as well as telephone follow‐up conducted every 6 months by trained research nurses. The end of follow‐up was defined as May 31, 2022. Patients were followed from the date of their first transthoracic echocardiography after MHD initiation until death, renal transplantation, loss to follow‐up, or the study end date, whichever occurred first. Loss to follow‐up was defined as inability to contact the patient or their family after at least three telephone attempts over two consecutive months. A total of nine patients (1.2%) were lost to follow‐up without documented death or transplantation and were censored at the time of last contact. The median follow‐up duration was 2.058 years (range 0.003–15.734 years).
4. Echocardiographic parameters: TTE was performed using standard two‐dimensional and Doppler echocardiography in all patients, with all measurements performed in accordance with the guidelines of the American Society of Echocardiography (ASE). All TTE examinations were performed after completion of a routine hemodialysis session (i.e., post‐dialysis), either on the same day (within 2–4 h after dialysis) or the following morning (after an overnight interdialytic interval), by three fixed board‐certified cardiologists who were blinded to the patients’ clinical data and study grouping. All scans were obtained in the post‐dialysis euvolemic state to minimize the acute confounding effects of intravascular volume overload on tricuspid regurgitation velocity (TRV), estimated pulmonary artery systolic pressure, and right‐sided chamber dimensions. Although the exact time interval between dialysis completion and TTE was not uniformly recorded in the retrospective database, the institutional protocol during the study period was to schedule elective echocardiography for MHD patients in the post‐dialysis window whenever possible. The presence of diastolic dysfunction, pericardial effusion, LVH, and aortic insufficiency was assessed via TTE. The following echocardiographic parameters were also measured: aortic root diameter, left atrial diameter (LAD), left ventricular end‐systolic diameter (LVESD), left ventricular end‐diastolic diameter (LVEDD), interventricular septal thickness (IVST), left ventricular posterior wall thickness (LVPWT), right ventricular diameter (RVD), right ventricular outflow tract (RVOT), main pulmonary artery diameter (MPAD), left ventricular fractional shortening (LVFS), left ventricular ejection fraction (LVEF), cardiac index (CI), stroke volume (SV), and left ventricular end‐diastolic volume (LVEDV).

### 2.3 Echocardiographic Classification of Echocardiographic Probability of Pulmonary Hypertension

Patients were stratified into three groups based on tricuspid regurgitation velocity (TRV) and additional echocardiographic signs of PH. According to the 2021 Chinese Guidelines for the Diagnosis and Treatment of Pulmonary Hypertension and the 2015 ESC/ERS Guidelines, additional echocardiographic signs supporting a diagnosis of PH include: (i) RVD enlargement and/or hypertrophy; (ii) right atrial (RA) enlargement; (iii) pulmonary artery dilation; (iv) reduced tricuspid annular plane systolic excursion (TAPSE); (v) inferior vena cava (IVC) dilation with reduced inspiratory collapse; (vi) flattening of the interventricular septum (D‐shaped left ventricle); and (vii) abnormal RVD outflow Doppler spectral pattern (e.g., mid‐systolic notching or shortened acceleration time).

In this retrospective cohort, the following additional signs were systematically evaluated from the archived TTE reports to classify PH probability: RVD dilation (increased RVD), RVD outflow tract enlargement (increased RVOT), main pulmonary artery dilation (increased MPAD), and left atrial enlargement (increased LAD) as indicators of right heart overload and pressure remodeling. Parameters such as TAPSE, IVC diameter/collapse, and septal flattening were not consistently documented in all historical echocardiograms and were therefore not uniformly incorporated into the probability stratification. Patients were classified as follows:

Low probability of PH: TRV ≤ 2.8 m/s with no additional echocardiographic signs of PH;

Intermediate probability of PH: TRV ≤ 2.8 m/s with additional echocardiographic signs of PH, or TRV of 2.9–3.4 m/s with no additional echocardiographic signs of PH;

High probability of PH: TRV of 2.9–3.4 m/s with additional echocardiographic signs of PH, or TRV > 3.4 m/s.

We recognize that volume status can substantially influence TRV and right heart parameters in MHD patients. By restricting TTE to the post‐dialysis setting, we aimed to standardize volume status as much as feasible within a retrospective framework. However, subtle residual volume differences may persist and could not be fully accounted for in this retrospective design.

Pulmonary artery systolic pressure (PASP) was estimated from the peak tricuspid regurgitation gradient using the simplified Bernoulli equation, combined with the estimated right atrial pressure (RAP), using the formula: PASP = 4 × (TRV)^2^ + RAP, where V is the peak velocity of the tricuspid regurgitation jet (m/s).

### 2.4 Definition of Study Endpoints and Competing Events

The primary event of interest was all‐cause mortality. Kidney transplantation was defined as the competing event, because it fundamentally alters the disease trajectory and precludes the observation of dialysis‐related mortality.

In the Fine‐Gray competing risk model, patients were coded as follows:

Event of interest (code = 1): patients who died from any cause during follow‐up.

Competing event (code = 2): patients who underwent kidney transplantation. These patients contributed person‐time from baseline until the date of transplantation, at which point they were removed from the risk set for mortality.

Censored (code = 0): patients who were alive without having received a kidney transplant at the end of the follow‐up period, as well as patients lost to follow‐up.

Importantly, patients who received a kidney transplant were not treated as censored; they were explicitly modeled as having experienced a competing event.

### 2.5 Statistical Analysis

A database was established using Microsoft Excel 2019. All statistical analyses were performed using IBM SPSS Statistics version 29.0 (IBM Corp., Armonk, NY, USA) and RStudio software version 2023.06.0 (R Foundation for Statistical Computing, Vienna, Austria). A two‐sided *p* value < 0.05 was considered statistically significant for all analyses.

For continuous variables, normally distributed data were presented as mean ± standard deviation (SD), and comparisons between two groups were performed using the independent samples *t*‐test for data with homogeneous variance. Non‐normally distributed continuous variables were presented as median (interquartile range, IQR), and comparisons between two groups were performed using the Mann‐Whitney *U* test. Categorical variables were presented as numbers (percentages), and comparisons between groups were performed using the chi‐square (χ^2^) test, or Fisher's exact test for cells with an expected count < 5. Handling of missing data. The proportion of missing values for each key variable was calculated. For the primary analyses, we performed complete‐case analysis; patients with missing data for any variable included in the Fine‐Gray model were excluded from that specific multivariable model. No imputation methods were applied. To assess potential bias due to missing data, we compared baseline characteristics (age, sex, PH probability group, and mortality status) between patients with complete data and those with any missing data using the chi‐square test (for categorical variables) or the independent *t*‐test/Mann‐Whitney *U* test (for continuous variables). A two‐sided *p* < 0.05 was considered to indicate a significant difference.

The Cox proportional hazards regression model was used to analyze factors associated with overall survival. The Kaplan‐Meier method with the log‐rank test was used to compare survival distributions between groups stratified by independent risk factors. In the presence of the competing risk of renal transplantation, the cumulative incidence function (CIF) was used to estimate the cumulative incidence of all‐cause mortality, with between‐group comparisons performed using Gray's test. The Fine‐Gray proportional subdistribution hazards model was used for multivariate competing risk regression analysis to identify independent predictors of all‐cause mortality in MHD patients.

## 3 Results

### 3.1 Survival Characteristics of MHD Patients in the Hemodialysis Center

#### 3.1.1 Clinical Outcomes of Enrolled Patients

A total of 758 patients receiving maintenance hemodialysis at our center between May 2010 and May 2022 were initially screened. Nine patients were excluded due to missing outcome data. Thus, the effective observational cohort comprised 749 patients, which served as the total population for the competing risk analysis.

Among these 749 patients, 64 patients underwent kidney transplantation during follow‐up, which was treated as the competing event. The remaining 685 patients were maintenance hemodialysis (MHD) patients who did not receive a kidney transplant. Within this MHD subgroup, 274 all‐cause deaths (event of interest) and 411 censored survivors (alive without transplantation at the end of follow‐up) were recorded. Therefore, the analytical cohort for the primary endpoint consisted of 749 individuals (Table 1).

**Table 1:** Baseline characteristics of the overall study population (*n* = 749).

Variable	Overall (*n* = 749)	Low echocardiographic probability of PH (*n* = 456)	Intermediate echocardiographic probability of PH (*n* = 218)	High echocardiographic probability of PH (*n* = 75)	*p* value
Age, years, median (range)	54.0 (18–80)	54.0 (41.0–63.0)	55.0 (44.0, 66.0)	54.0 (42.0, 62.0)	0.154
Male, *n* (%)	478 (63.82)	297 (65.13)	132 (60.55)	49 (65.33)	0.491
Smoking history, *n* (%)	183 (24.43)	117 (25.66)	44 (20.18)	22 (29.33)	0.128
Treatment duration, months, median (range)	32.0 (3–133)	32.0 (3–133)	34.0 (15.0, 58.0)	31.0 (12.0, 55.0)	0.181
**Underlying diseases, *n* (%)**					0.013*
Chronic nephritis	510 (68.09)	319 (69.96)	142 (65.14)	49 (65.33)	
Diabetes mellitus	80 (10.68)	37 (8.11)	35 (16.06)	8 (10.67)	
Hypertension	79 (10.55)	46 (10.09)	19 (8.72)	14 (18.67)	
Obstructive nephropathy	39 (5.21)	27 (5.92)	10 (4.59)	2 (2.67)	
Polycystic kidney disease	29 (3.87)	20 (4.39)	7 (3.21)	2 (2.67)	
Gouty nephropathy	7 (0.93)	6 (1.32)	1 (0.46)	0 (0.00)	
Multiple myeloma	5 (0.67)	1 (0.22)	4 (1.83)	0 (0.00)	
**Complications, *n* (%)**					
Anemia	671 (89.59)	410 (89.91)	192 (88.07)	69 (92.00)	0.590
Hypertension	447 (59.68)	276 (60.53)	122 (55.96)	49 (65.33)	0.304
Coronary artery disease	47 (6.28)	23 (5.04)	17 (7.80)	7 (9.33)	0.199
Diastolic dysfunction	247 (32.98)	165 (36.18)	67 (30.73)	15 (20.00)	0.016*
Pericardial effusion	183 (24.43)	102 (22.37)	67 (30.73)	14 (18.67)	0.029*
Left ventricular hypertrophy	377 (50.33)	226 (49.56)	123 (56.42)	28 (37.33)	0.015*
Aortic insufficiency	265 (35.38)	126 (27.63)	109 (50.00)	30 (40.00)	< 0.001*
**Vascular access, *n* (%)**					< 0.001*
TCC	254 (33.91)	183 (40.13)	62 (28.44)	9 (12.00)	
AVF/AVG	495 (66.09)	273 (59.87)	156 (71.56)	66 (88.00)	
**Survival outcome**					< 0.001*
Survival, *n* (%)	413 (55.1)	287 (62.94)	104 (47.71)	22 (29.33)	
Mortality, *n* (%)	272 (36.3)	124 (27.19)	104 (47.71)	44 (58.67)	
Renal transplantation, *n* (%)	64 (8.5)	45 (9.87)	10 (4.59)	9 (12.00)	
Survival time, years, median (range)	2.058 (0.003–15.734)	2.350 (0.003–15.734)	1.873 (0.7, 4.2)	1.529 (0.1, 3.0)	< 0.001*

Abbreviations: AVF, Arteriovenous Fistula; AVG, Arteriovenous Graft; PH, Pulmonary Hypertension; TCC, Tunneled Cuffed Catheter.

#### 3.1.2 Overall Clinical Characteristics of the Observational Cohort

A total of 685 MHD patients treated in our center from May 2010 to May 2022 were included in this study, of whom 436 (63.65%) were male, with a median age of 55 (18–85) years. The mean follow‐up duration was 2.058 (0.003–15.734) years, and the median overall survival was 7.279 years.

Missing data: among the 749 included patients, complete data for all demographic and echocardiographic variables were available for all patients (0% missing). For laboratory parameters, the number and proportion of missing values were as follows: serum albumin: 43/749 (5.7%); hemoglobin: 38/749 (5.1%); serum phosphate: 52/749 (6.9%); C‐reactive protein (CRP): 84/749 (11.2%). Systolic blood pressure was missing for 31/749 patients (4.1%). Cause of kidney failure was documented for all patients. Details of missing data for each variable used in the multivariable models are presented in Table S1. Patients with complete data (*n* = 628 for the fully adjusted model) did not differ significantly from those with any missing data (*n* = 121) with respect to age (53.1 ± 14.2 vs. 52.4 ± 15.0 years, *p* = 0.61), sex (63.9% vs. 62.8% male, *p* = 0.82), PH probability distribution (*p* = 0.73), or all‐cause mortality during follow‐up (36.1% vs. 38.0%, *p* = 0.67).

Among the 685 MHD patients, the primary underlying kidney diseases were as follows: chronic nephritis in 473 (69.05%) cases, diabetic kidney disease in 73 (10.66%) cases, hypertensive nephropathy in 73 (10.66%) cases, obstructive nephropathy in 27 (3.94%) cases, polycystic kidney disease in 27 (3.94%) cases, interstitial nephritis in 7 (1.02%) cases, and multiple myeloma in 5 (0.73%) cases. The median treatment duration of the cohort was 32 months (range 3–133 months).

Echocardiographic findings showed that 230 (33.58%) patients had diastolic dysfunction, 165 (24.09%) had pericardial effusion, 349 (50.95%) had LVH, and 243 (35.47%) had aortic insufficiency. Regarding tricuspid regurgitation (TR), 66 (9.64%) patients had severe TR, 208 (30.36%) had moderate TR, and 411 (60.00%) had mild or no TR. For vascular access, 227 (33.14%) patients used a tunneled cuffed catheter (TCC), and 458 (66.86%) used an AVF or arteriovenous graft (AVG) (Table 2).

**Table 2:** Comparison of baseline clinical characteristics between the survival and mortality groups (*n* = 685).

Variable	Survival (*n* = 411) Outcome (%/mean ± SD)	Mortality (*n* = 274) Outcome (%/mean ± SD)	Total	χ^2^/t value	*p* value
Gender, *n* (%)	256 (62.29)	180 (65.69)	436 (63.65)	0.824	0.364
Smoking history, *n* (%)	111 (27.01)	60 (21.90)	171 (24.96)	2.291	0.13
Underlying diseases, *n* (%)					
Chronic nephritis	292 (71.05)	181 (66.06)	473 (69.05)	11.916	0.064
Diabetes mellitus	37 (9.00)	36 (13.14)	73 (10.66)		
Hypertension	41 (9.98)	32 (11.68)	73 (10.66)		
Obstructive nephropathy	20 (4.87)	7 (2.55)	27 (3.94)		
Polycystic kidney disease	14 (3.41)	13 (4.74)	27 (3.94)		
Gouty nephropathy	6 (1.46)	1 (0.36)	7 (1.02)		
Multiple myeloma	1 (0.24)	4 (1.46)	5 (0.73)		
Anemia, *n* (%)	373 (90.75)	240 (87.59)	613 (89.49)	1.749	0.186
Hypertension, *n* (%)	250 (60.83)	159 (58.03)	409 (59.71)	0.535	0.465
Coronary artery disease, *n* (%)	23 (5.60)	21 (7.66)	44 (6.42)	1.17	0.279
Left ventricular hypertrophy, *n* (%)	220 (53.53)	129 (47.08)	349 (50.95)	2.735	0.098
Diastolic dysfunction, *n* (%)	138 (33.58)	92 (33.58)	230 (33.58)	0	1
Pericardial effusion, *n* (%)	96 (23.36)	69 (25.18)	165 (24.09)	0.299	0.584
Aortic insufficiency, *n* (%)	130 (31.63)	113 (41.24)	243 (35.47)	6.634	0.010*
Survival time, years	3.90 ± 2.74	1.95 ± 2.51	3.12 ± 2.815	9.598	0.000**
Age, years	50.62 ± 13.84	56.32 ± 14.50	52.90 ± 14.37	−5.187	0.000**
Treatment duration, months	37.46 ± 30.50	36.11 ± 27.31	36.92 ± 29.25	0.592	0.554
LAD, mm	37.81 ± 6.80	40.13 ± 6.98	38.74 ± 6.96	−4.317	0.000**
LVEDD, mm	53.47 ± 7.23	54.66 ± 7.25	53.94 ± 7.26	−2.1	0.036*
LVESD, mm	34.43 ± 7.43	36.12 ± 8.16	35.11 ± 7.77	−2.792	0.005**
IVST, mm	12.45 ± 1.56	12.72 ± 1.55	12.56 ± 1.56	−2.217	0.027*
LVPWT, mm	12.27 ± 1.49	12.38 ± 1.60	12.31 ± 1.54	−0.881	0.379
RVD, mm	20.17 ± 2.75	20.94 ± 2.88	20.47 ± 2.83	−3.524	0.000**
RVOT, mm	28.35 ± 3.34	28.80 ± 3.41	28.53 ± 3.38	−1.71	0.088
MPAD, mm	26.42 ± 3.27	27.07 ± 3.34	26.68 ± 3.31	−2.502	0.013*
LVFS, %	0.64 ± 0.10	0.63 ± 0.11	0.64 ± 0.11	2.025	0.043*
LVEF, %	35.53 ± 8.26	34.56 ± 8.51	35.14 ± 8.37	1.489	0.137
SV, ml	90.30 ± 24.19	90.84 ± 22.51	90.51 ± 23.52	−0.297	0.767
CI, L/min	7.32 ± 2.26	7.34 ± 2.10	7.33 ± 2.20	−0.117	0.907
LVEDV, mL	144.40 ± 42.81	147.26 ± 41.96	145.55 ± 42.46	−0.865	0.388
Echocardiographic probability of PH grouping, *n* (%)					
Low probability	287 (69.83)	124 (45.26)	411 (60.00)	49.466	0.000**
Intermediate probability	104 (25.30)	104 (37.96)	208 (30.36)		
High probability	20 (4.87)	46 (16.79)	66 (9.64)		
Vascular access, *n* (%)					
TCC	143 (34.79)	84 (30.66)	227 (33.14)	1.269	0.26
AVF/AVG	268 (65.21)	190 (69.34)	458 (66.86)		

Abbreviations: AVF, Arteriovenous Fistula; AVG, Arteriovenous Graft; CI, Cardiac Index; IVST, Interventricular Septal Thickness; LAD, Left Atrial Diameter; LVEDD, Left Ventricular End‐Diastolic Diameter; LVEDV, Left Ventricular End‐Diastolic Volume; LVEF, Left Ventricular Ejection Fraction; LVESD, Left Ventricular End‐Systolic Diameter; LVFS, Left Ventricular Fractional Shortening; LVPWT, Left Ventricular Posterior Wall Thickness; MPAD, Main Pulmonary Artery Diameter; PH, Pulmonary Hypertension; SD, Standard Deviation; SV, Stroke Volume; TCC, Tunneled Cuffed Catheter.

#### 3.1.3 Baseline Clinical Characteristics of MHD Patients With TCC and AVF/AVG Access

A total of 227 MHD patients with TCC access treated in our center from May 2010 to May 2022 were included. Among them, 137 (60.35%) were male, with a mean age of 52.06 ± 14.13 years, and a mean follow‐up duration of 3.24 ± 3.01 years. The primary underlying kidney diseases in the TCC cohort were chronic nephritis in 162 (71.37%) cases, diabetic kidney disease in 30 (13.22%) cases, hypertensive nephropathy in 18 (7.93%) cases, obstructive nephropathy in 8 (3.52%) cases, polycystic kidney disease in 5 (2.20%) cases, interstitial nephritis in 3 (1.32%) cases, and multiple myeloma in 1 (0.44%) case. The median treatment duration was 37 months (range 3–133 months). In this cohort, 143 (63.00%) patients survived, 84 (37.00%) died, 56 (24.67%) had a smoking history, 206 (90.75%) had anemia, 145 (63.88%) had hypertension, and 14 (6.17%) had coronary artery disease.

A total of 458 MHD patients with AVF/AVG access treated during the same period were included. Among them, 299 (65.28%) were male, with a mean survival time of 3.17 ± 2.93 years and a mean treatment duration of 36.83 months. The primary underlying kidney diseases in the AVF/AVG cohort were chronic nephritis in 311 (67.90%) cases, diabetic kidney disease in 43 (9.39%) cases, hypertensive nephropathy in 55 (12.01%) cases, obstructive nephropathy in 19 (4.15%) cases, polycystic kidney disease in 22 (4.80%) cases, interstitial nephritis in 4 (0.87%) cases, and multiple myeloma in 4 (0.87%) cases. The median treatment duration was 36 months (range 3–133 months). In this cohort, 270 (58.95%) patients survived, 188 (41.05%) died, 115 (25.11%) had a smoking history, 407 (88.86%) had anemia, 264 (57.64%) had hypertension, and 30 (6.55%) had coronary artery disease.

No statistically significant differences were observed in baseline clinical data between the TCC and AVF/AVG cohorts (Table 3).

**Table 3:** Comparison of baseline data between patients with two types of vascular access (*n* = 685).

Variable	TCC (*n* = 227) mean ± SD/*n* (%)	AVF (*n* = 458) mean ± SD/*n* (%)	*p* value
Survival time, years	3.24 ± 3.01	3.17 ± 2.93	0.767
Treatment duration, months	37.11 ± 30.97	36.83 ± 28.40	0.909
Age, years	52.06 ± 14.13	53.31 ± 14.48	0.281
Survival outcome, *n* (%)			
Survival	143 (63.00)	270 (58.95)	0.309
Mortality	84 (37.00)	188 (41.05)	
Male gender, *n* (%)	137 (60.35)	299 (65.28)	0.207
Smoking history, *n* (%)	56 (24.67)	115 (25.11)	0.9
Underlying diseases, *n* (%)			
Chronic nephritis	162 (71.37)	311 (67.90)	0.226
Diabetes mellitus	30 (13.22)	43 (9.39)	
Hypertension	18 (7.93)	55 (12.01)	
Obstructive nephropathy	8 (3.52)	19 (4.15)	
Polycystic kidney disease	5 (2.20)	22 (4.80)	
Gouty nephropathy	3 (1.32)	4 (0.87)	
Multiple myeloma	1 (0.44)	4 (0.87)	
Anemia, *n* (%)	206 (90.75)	407 (88.86)	0.449
Hypertension, *n* (%)	145 (63.88)	264 (57.64)	0.117
Coronary artery disease, *n* (%)	14 (6.17)	30 (6.55)	0.847

Abbreviations: AVF, Arteriovenous Fistula; SD, Standard Deviation; TCC, Tunneled Cuffed Catheter.

### 3.2 Survival Analysis of MHD Patients Using the Competing Risk Model

#### 3.2.1 Clinical Outcomes Based on the Competing Risk Model

Among the 749 patients in the analytic cohort, all‐cause death (*n* = 274) was defined as the event of interest, and kidney transplantation (*n* = 64) was defined as the competing event. According to the Fine‐Gray model, patients who received a kidney transplant were coded as having experienced the competing event and were removed from the mortality risk set at the time of transplantation. Patients who were alive without having received a kidney transplant at the end of follow‐up (*n* = 411) were treated as censored. No patients were lost to follow‐up.

#### 3.2.2 Factors Associated With Cumulative Mortality Incidence in MHD Patients

After adjusting for competing risks, the cumulative incidence of all‐cause mortality in MHD patients was significantly associated with PH probability stratification, age, aortic insufficiency, LAD, LVEDD, LVESD, RVD, MPAD, and LVEF (Gray's test *p* < 0.05) (Table 4).

**Table 4:** Univariate competing risk model (*n* = 749).

Variables	Regression coefficient (b)	Standard Error (SE)	HR (95%CI)	z value	*p* value
Echocardiographic probability of PH grouping					
0: Low probability	Ref	—	—	—	—
1: Intermediate probability	0.505	0.124	1.658 (1.301, 2.113)	4.082	< 0.001**
2: High probability	0.872	0.160	2.391 (1.748, 3.271)	5.455	< 0.001**
Vascular access	0.172	0.128	1.187 (0.923, 1.527)	1.338	0.18
Gender	−0.095	0.126	0.909 (0.711, 1.163)	−0.758	0.45
Age, years	0.024	0.005	1.024 (1.014, 1.034)	4.862	< 0.001**
Treatment duration, months	−0.001	0.002	0.999 (0.995, 1.003)	−0.417	0.68
Smoking history	−0.131	0.145	0.878 (0.660, 1.166)	−0.900	0.37
Underlying diseases	0.003	0.047	1.003 (0.914, 1.101)	0.069	0.94
Anemia	−0.259	0.187	0.772 (0.535, 1.115)	−1.381	0.17
Hypertension	−0.072	0.121	0.930 (0.733, 1.180)	−0.596	0.55
Coronary artery disease	0.123	0.218	1.131 (0.737, 1.735)	0.564	0.57
Left ventricular hypertrophy	−0.203	0.119	0.816 (0.646, 1.032)	−1.699	0.089
Diastolic dysfunction	0.098	0.126	1.103 (0.861, 1.412)	0.775	0.44
Pericardial effusion	0.045	0.139	1.046 (0.797, 1.373)	0.324	0.75
Aortic insufficiency	0.294	0.120	1.341 (1.060, 1.697)	2.446	0.014*
LAD, mm	0.037	0.009	1.038 (1.020, 1.055)	4.341	< 0.001**
LVEDD, mm	0.019	0.008	1.020 (1.003, 1.036)	2.331	0.02*
LVESD, mm	0.019	0.007	1.019 (1.004, 1.034)	2.549	0.011*
IVST, mm	0.065	0.035	1.067 (0.995, 1.144)	1.831	0.067
LVPWT, mm	0.015	0.040	1.015 (0.938, 1.098)	0.373	0.71
RVD, mm	0.082	0.019	1.085 (1.046, 1.125)	4.388	< 0.001**
RVOT, mm	0.029	0.017	1.029 (0.996, 1.064)	1.714	0.086
MPAD, mm	0.046	0.018	1.047 (1.011, 1.084)	2.602	0.009**
LVEF, %	−1.161	0.565	0.313 (0.104, 0.947)	−2.057	0.04*
LVFS, %	−0.010	0.007	0.990 (0.977, 1.004)	−1.435	0.15
CI, L/min	0.031	0.027	1.031 (0.978, 1.088)	1.132	0.26
LVEDV, mL	0.002	0.001	1.002 (0.999, 1.005)	1.530	0.13
SV, mL	0.003	0.002	1.003 (0.998, 1.008)	1.065	0.29

Abbreviations: b, Regression Coefficient; CI, Cardiac Index; HR, Hazard Ratio; IVST, Interventricular Septal Thickness; LAD, Left Atrial Diameter; LVEDD, Left Ventricular End‐Diastolic Diameter; LVEDV, Left Ventricular End‐Diastolic Volume; LVEF, Left Ventricular Ejection Fraction; LVESD, Left Ventricular End‐Systolic Diameter; LVFS, Left Ventricular Fractional Shortening; LVPWT, Left Ventricular Posterior Wall Thickness; MPAD, Main Pulmonary Artery Diameter; PH, Pulmonary Hypertension; Ref, Reference Group; RVD, Right Ventricular Diameter; RVOT, Right Ventricular Outflow Tract; SE, Standard Error; SV, Stroke Volume; 95% CI, 95% Confidence Interval.

**Figure 1:**
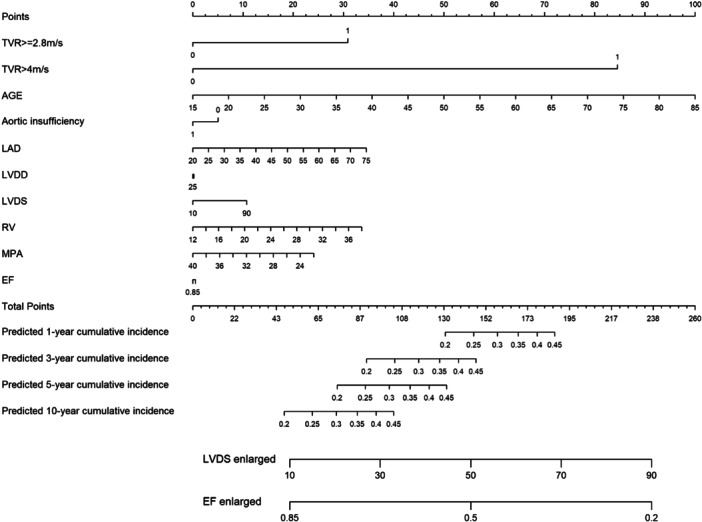
Nomogram predicting the 1‐, 3‐, 5‐, and 10‐year cumulative incidence of all‐cause mortality in maintenance hemodialysis patients, developed using a competing risk model that accounts for kidney transplantation as a competing event. The nomogram integrates the following predictors: pulmonary hypertension probability groups (intermediate: TRV 2.9–3.4 m/s; high: TRV > 3.4 m/s), age, aortic insufficiency, left atrial diameter (LAD), left ventricular end‐diastolic diameter (LVEDD), left ventricular end‐systolic diameter (LVESD), right ventricular diameter (RV), main pulmonary artery diameter (MPAD), and left ventricular ejection fraction (LVEF). To use the nomogram, locate each predictor on its corresponding axis, draw a vertical line to the “Points” scale to obtain the individual point contribution. Sum the points for all predictors to obtain the “Total Points”. Draw a vertical line from the “Total Points” scale to each predicted outcome scale to estimate the 1‐, 3‐, 5‐, and 10‐year cumulative incidence of mortality.

The Fine‐Gray competing risk model was constructed for multivariate analysis. Nine variables with *p* < 0.1 in the univariate analysis were entered into the multivariate model, including PH probability stratification, age, aortic insufficiency, LAD, LVEDD, LVESD, RVD, MPAD, and LVEF. The results of the multivariate analysis showed that intermediate probability of PH (hazard ratio [HR] = 1.871, 95% confidence interval [CI] 1.419–2.468, *p* < 0.001), high probability of PH (HR = 2.877, 95% CI 2.029–4.079, *p* < 0.001), age (HR = 1.023, 95% CI 1.012–1.033, *p* < 0.001), LAD (HR = 1.022, 95% CI 1.001–1.044, *p* = 0.039), and RVD (HR = 1.049, 95% CI 1.007–1.093, *p* = 0.021) were independent risk factors for all‐cause mortality in MHD patients (Table 5). Based on the competing risk model, a nomogram was constructed to predict mortality in maintenance hemodialysis patients in the presence of kidney transplantation as a competing risk. This nomogram integrates pulmonary hypertension grading, cardiac structural remodeling indicators (particularly right ventricular diameter), and traditional risk factors (age), thereby establishing a precise predictive model that reflects the real‐world scenario of hemodialysis patients (Figure 1).

**Table 5:** Multivariate competing risk model (*n* = 749).

Variables	Regression coefficient (b)	Standard Error (SE)	HR (95% CI)	z value	*p* value
Echocardiographic probability of PH grouping					
0: Low probability	Ref	—	—	—	—
1: Intermediate probability	0.627	0.141	1.871 (1.419, 2.468)	4.439	< 0.001**
2: High probability	1.057	0.178	2.877 (2.029, 4.079)	5.932	< 0.001**
Age, years	0.023	0.005	1.023 (1.012, 1.033)	4.335	< 0.001**
Aortic insufficiency	−0.009	0.131	0.991 (0.767, 1.280)	−0.071	0.94
LAD, mm	0.022	0.011	1.022 (1.001, 1.044)	2.064	0.039*
LVEDD, mm	−0.006	0.017	0.994 (0.962, 1.027)	−0.362	0.72
LVESD, mm	0.000	0.022	1.000 (0.957, 1.044)	−0.019	0.98
RVD, mm	0.048	0.021	1.049 (1.007, 1.093)	2.299	0.021*
MPAD, mm	−0.015	0.020	0.986 (0.947, 1.025)	−0.721	0.47
LVEF, %	−0.353	1.081	0.703 (0.084, 5.842)	−0.327	0.74

Abbreviations: b, Regression Coefficient; HR, Hazard Ratio; LAD, Left Atrial Diameter; LVEDD, Left Ventricular End‐Diastolic Diameter; LVEF, Left Ventricular Ejection Fraction; LVESD, Left Ventricular End‐Systolic Diameter; MPAD, Main Pulmonary Artery Diameter; PH, Pulmonary Hypertension; Ref, Reference Group; RVD, Right Ventricular Diameter; SE, Standard Error; 95% CI, 95% Confidence Interval.

### 3.3 Cumulative Incidence of All‐Cause Mortality Accounting for Kidney Transplantation as a Competing Event

**Figure 2:**
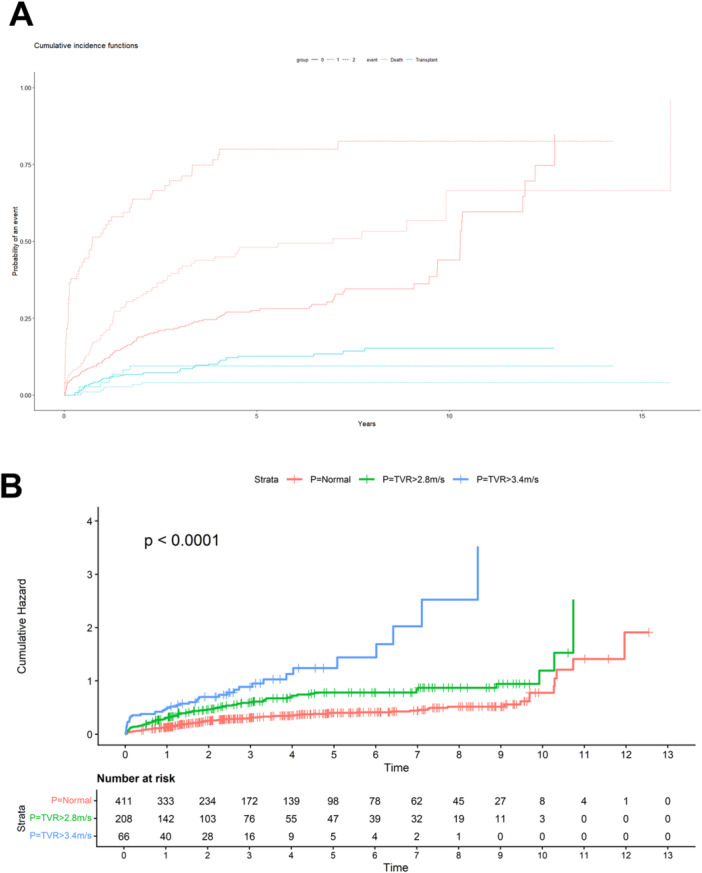
Cumulative incidence of all‐cause mortality in maintenance hemodialysis patients. (A) Overall cohort (*n* = 749). The subdistribution cumulative incidence function (CIF) for mortality (solid line) and kidney transplantation (dashed line) was estimated using the Fine‐Gray competing risk model. This CIF represents the actual probability of experiencing the event while accounting for the competing risk of kidney transplantation, and is mathematically distinct from the cumulative hazard (which sums hazard rates over time regardless of competing events) and from 1 minus the Kaplan‐Meier survival function (which treats competing events as censored and therefore overestimates the marginal probability of mortality). (B) Cumulative incidence of mortality stratified by echocardiographic probability of PH groups based on tricuspid regurgitant velocity (TRV): low probability (TRV ≤ 2.8 m/s), intermediate probability (TRV 2.9–3.4 m/s), and high probability (TRV > 3.4 m/s). Gray's test was used for between‐group comparisons.

Using the Fine‐Gray subdistribution cumulative incidence function (CIF)—which estimates the actual probability of death while accounting for renal transplantation as a competing event, and differs from the cumulative hazard or 1‐Kaplan‐Meier estimates—the cumulative incidence of mortality increased progressively with longer dialysis vintage, reaching 15.0% at 1 year, 30.0% at 3 years, 38.0% at 5 years, and 55.0% at 10 years. The cumulative incidence of kidney transplantation, the competing event, was 2.0% at 1 year and increased to 11.0% by 10 years, indicating that a proportion of patients were removed from the mortality risk set due to receiving a transplant (Figure 2A). A clear gradient in mortality risk was observed across the three groups throughout the follow‐up period. The high‐probability PH group (TRV > 3.4 m/s) exhibited the highest cumulative mortality, followed by the intermediate‐probability group (TRV 2.9–3.4 m/s), while the low‐probability group (TRV ≤ 2.8 m/s) demonstrated the lowest mortality risk. At 1 year, the cumulative mortality incidence was 11.3% in the low‐probability group, 25.5% in the intermediate‐probability group, and 34.7% in the high‐probability group. By 5 years, these estimates increased to 29.4%, 51.8%, and 63.1%, respectively. At 10 years, the cumulative mortality reached 47.4% in the low‐probability group, 67.5% in the intermediate‐probability group, and 87.5% in the high‐probability group. Gray's test confirmed a statistically significant difference in cumulative mortality incidence among the three PH probability groups (Figure 2B). These findings demonstrate that PH probability is a strong predictor of long‐term mortality in maintenance hemodialysis patients, with risk differences emerging early and widening over time, even after accounting for the competing risk of kidney transplantation.

### 3.4 Survival Rates of MHD Patients

The overall 1‐year, 3‐year, 5‐year, and 10‐year all‐cause survival rates of MHD patients in our center were 82.22%, 67.71%, 60.60%, and 42.22%, respectively. Stratified by PH probability:

In the low PH probability group, the 1‐year, 3‐year, 5‐year, and 10‐year survival rates were 88.71%, 76.36%, 70.64%, and 52.58%, respectively;

In the intermediate PH probability group, the 1‐year, 3‐year, 5‐year, and 10‐year survival rates were 74.49%, 56.93%, 48.21%, and 32.49%, respectively;

In the high PH probability group, the 1‐year, 3‐year, 5‐year, and 10‐year survival rates were 65.33%, 47.78%, 36.94%, and 12.54%, respectively (Table 6).

**Table 6:** Survival rates of MHD patients in our center (*n* = 749).

Groups	1‐year survival rate	3‐year survival rate	5‐year survival rate	10‐year survival rate
Overall	82.22%	67.71%	60.60%	42.22%
Low Echocardiographic probability of PH	88.71%	76.36%	70.64%	52.58%
Intermediate Echocardiographic probability of PH	74.49%	56.93%	48.21%	32.49%
High Echocardiographic probability of PH	65.33%	47.78%	36.94%	12.54%

Abbreviations: MHD, Maintenance Hemodialysis; PH, Pulmonary Hypertension.

### 3.5 Survival Analysis of MHD Patients With TCC Access Using the Competing Risk Model

#### 3.5.1 Clinical Outcomes of MHD Patients in the TCC Cohort

A total of 254 MHD patients with TCC access were included in the competing risk model analysis. All‐cause mortality was set as the event of interest, while renal transplantation (n = 27) was treated as the competing event. As of the end of the follow‐up period, 143 patients remained alive, which were handled as censored data according to the competing risk model principles.

#### 3.5.2 Factors Associated With Cumulative Mortality Incidence in the TCC Cohort

After adjusting for competing risks, the cumulative incidence of all‐cause mortality in TCC‐treated MHD patients was significantly associated with PH probability stratification, age, LAD, LVPWT, LVESV, and SV (Gray's test *p* < 0.05) (Table 7).

**Table 7:** Univariate competing risk model for patients with TCC (Access = 0, *n* = 254).

Variables	Regression coefficient (b)	Standard Error (SE)	HR (95% CI)	z value	*p* value
Echocardiographic probability of PH grouping					
0: Low probability	Ref	—	—	—	—
1: Intermediate probability	0.564	0.229	1.758 (1.123, 2.751)	2.469	0.014*
2: High probability	1.580	0.370	4.856 (2.349, 10.038)	4.265	< 0.001**
Gender	0.067	0.219	1.070 (0.697, 1.642)	0.309	0.76
Age, years	0.023	0.009	1.023 (1.006, 1.041)	2.645	0.008**
Treatment duration, months	0.001	0.003	1.001 (0.994, 1.007)	0.162	0.87
Smoking history	−0.515	0.288	0.597 (0.340, 1.050)	−1.789	0.074
Underlying diseases	0.022	0.080	1.022 (0.873, 1.196)	0.268	0.79
Anemia	−0.536	0.359	0.585 (0.289, 1.184)	−1.491	0.14
Hypertension	−0.133	0.221	0.876 (0.568, 1.350)	−0.600	0.55
Coronary artery disease	0.290	0.448	1.336 (0.556, 3.214)	0.648	0.52
Left ventricular hypertrophy	−0.175	0.215	0.840 (0.551, 1.279)	−0.813	0.42
Diastolic dysfunction	0.228	0.218	1.256 (0.819, 1.924)	1.045	0.30
Pericardial effusion	−0.138	0.245	0.871 (0.539, 1.407)	−0.565	0.57
Mitral insufficiency	0.159	0.215	1.172 (0.769, 1.785)	0.738	0.46
Aortic insufficiency	0.381	0.215	1.464 (0.961, 2.231)	1.776	0.076
LAD, mm	0.055	0.016	1.056 (1.024, 1.089)	3.517	< 0.001**
LVEDD, mm	0.019	0.015	1.020 (0.989, 1.051)	1.247	0.21
LVESD, mm	0.015	0.014	1.015 (0.988, 1.043)	1.079	0.28
IVST, mm	0.123	0.071	1.131 (0.983, 1.301)	1.721	0.085
LVPWT, mm	0.189	0.079	1.208 (1.035, 1.409)	2.397	0.017*
RVD, mm	0.052	0.040	1.053 (0.973, 1.139)	1.279	0.20
RVOT, mm	0.056	0.030	1.057 (0.997, 1.121)	1.863	0.063
MPAD, mm	0.020	0.033	1.020 (0.956, 1.089)	0.599	0.55
LVEF, %	−0.764	1.164	0.466 (0.048, 4.563)	−0.656	0.51
LVFS, %	0.000	0.016	1.000 (0.969, 1.032)	0.003	> 0.999
CI, L/min	0.050	0.054	1.052 (0.946, 1.169)	0.931	0.35
LVEDV, mL	0.006	0.003	1.006 (1.000, 1.011)	2.063	0.039*
SV, mL	0.010	0.005	1.010 (1.000, 1.020)	1.990	0.047*

Abbreviations: b, Regression Coefficient; CI, Cardiac Index; HR, Hazard Ratio; IVST, Interventricular Septal Thickness; LAD, Left Atrial Diameter; LVEDD, Left Ventricular End‐Diastolic Diameter; LVEDV, Left Ventricular End‐Diastolic Volume; LVEF, Left Ventricular Ejection Fraction; LVESD, Left Ventricular End‐Systolic Diameter; LVFS, Left Ventricular Fractional Shortening; LVPWT, Left Ventricular Posterior Wall Thickness; MPAD, Main Pulmonary Artery Diameter; PH, Pulmonary Hypertension; Ref, Reference Group; RVD, Right Ventricular Diameter; RVOT, Right Ventricular Outflow Tract; SE, Standard Error; SV, Stroke Volume; TCC, Tunneled Cuffed Catheter; 95% CI, 95% Confidence Interval.

The Fine‐Gray model was used for multivariate analysis. Six variables with *p* < 0.1 in the univariate analysis were entered into the model, including PH probability stratification, age, LAD, LVPWT, LVESV, and SV. Multivariate analysis revealed that high probability of PH (HR = 4.979, 95% CI 2.435–10.18, *p* < 0.001) and age (HR = 1.021, 95% CI 1.003–1.038, *p* = 0.021) were independent risk factors for all‐cause mortality in TCC‐treated MHD patients (Table 8).

**Table 8:** Multivariate competing risk model for patients with TCC (Access = 0, *n* = 254).

Variables	Regression coefficient (b)	Standard Error (SE)	HR (95% CI)	z value	*p* value
Echocardiographic probability of PH grouping					
0: Low probability	Ref	—	—	—	—
1: Intermediate probability	0.490	0.263	1.633 (0.975, 2.734)	1.863	0.063
2: High probability	1.605	0.365	4.979 (2.435, 10.180)	4.399	< 0.001**
Age, years	0.020	0.009	1.021 (1.003, 1.038)	2.309	0.021*
LAD, mm	0.028	0.016	1.028 (0.997, 1.061)	1.757	0.079
LVPWT, mm	0.158	0.094	1.171 (0.973, 1.408)	1.672	0.095
LVEDV, mL	−0.001	0.005	0.999 (0.990, 1.009)	−0.118	0.91
SV, mL	0.004	0.009	1.004 (0.987, 1.022)	0.503	0.62

Abbreviations: b, Regression Coefficient; HR, Hazard Ratio; LAD, Left Atrial Diameter; LVEDV, Left Ventricular End‐Diastolic Volume; LVPWT, Left Ventricular Posterior Wall Thickness; PH, Pulmonary Hypertension; Ref, Reference Group; SE, Standard Error; SV, Stroke Volume; TCC, Tunneled Cuffed Catheter; 95% CI: 95% Confidence Interval.

### 3.6 Competing Risk Model Analysis of Risk Factors for All‐Cause Mortality in MHD Patients With AVF/AVG Access

#### 3.6.1 Clinical Outcomes of MHD Patients in the AVF/AVG Cohort

A total of 495 MHD patients with AVF/AVG access were included in the competing risk model analysis. All‐cause mortality was set as the event of interest, while renal transplantation (*n* = 37) was treated as the competing event. As of the end of the follow‐up period, 268 patients remained alive, which were handled as censored data in line with the competing risk model principles.

#### 3.6.2 Factors Associated With Cumulative Mortality Incidence in the AVF/AVG Cohort

After adjusting for competing risks, the cumulative incidence of all‐cause mortality in AVF/AVG‐treated MHD patients was significantly associated with PH probability stratification, age, LAD, LVESD, RVD, MPAD, and LVEF (Gray's test *p* < 0.05) (Table 9).

**Table 9:** Univariate competing risk model for patients with AVF/AVG (Access = 1, *n* = 495).

Variables	Regression coefficient (b)	Standard Error (SE)	HR (95% CI)	z value	*p* value
Echocardiographic probability of PH grouping					
0: Low probability	Ref	—	—	—	—
1: Intermediate probability	0.460	0.148	1.584 (1.185, 2.117)	3.109	0.002**
2: High probability	0.724	0.179	2.064 (1.454, 2.928)	4.058	< 0.001**
Gender	−0.160	0.153	0.852 (0.631, 1.150)	−1.045	0.30
Age, years	0.024	0.006	1.024 (1.012, 1.036)	4.026	< 0.001**
Treatment duration, months	−0.002	0.002	0.998 (0.993, 1.003)	−0.726	0.47
Smoking history	0.017	0.169	1.017 (0.731, 1.415)	0.099	0.92
Underlying diseases	−0.006	0.058	0.994 (0.887, 1.114)	−0.101	0.92
Anemia	−0.135	0.219	0.873 (0.569, 1.341)	−0.619	0.54
Hypertension	−0.039	0.145	0.962 (0.724, 1.277)	−0.269	0.79
Coronary artery disease	0.050	0.250	1.051 (0.644, 1.716)	0.200	0.84
Left ventricular hypertrophy	−0.204	0.145	0.816 (0.614, 1.083)	−1.406	0.16
Diastolic dysfunction	0.035	0.155	1.035 (0.764, 1.403)	0.225	0.82
Pericardial effusion	0.138	0.170	1.148 (0.823, 1.603)	0.814	0.42
Mitral insufficiency	0.220	0.144	1.246 (0.941, 1.651)	1.533	0.13
Aortic insufficiency	0.237	0.144	1.268 (0.955, 1.682)	1.643	0.10
LAD, mm	0.030	0.010	1.030 (1.010, 1.051)	2.996	0.003**
LVEDD, mm	0.019	0.010	1.019 (0.999, 1.039)	1.881	0.060
LVESD, mm	0.020	0.009	1.020 (1.003, 1.038)	2.257	0.024*
IVST, mm	0.038	0.040	1.039 (0.960, 1.124)	0.943	0.35
LVPWT, mm	−0.049	0.044	0.952 (0.873, 1.038)	−1.106	0.27
RVD, mm	0.086	0.021	1.090 (1.045, 1.136)	4.010	< 0.001**
RVOT, mm	0.014	0.021	1.014 (0.973, 1.056)	0.660	0.51
MPAD, mm	0.053	0.021	1.054 (1.012, 1.098)	2.547	0.011*
LVEF, %	−1.262	0.634	0.283 (0.082, 0.980)	−1.992	0.046*
LVFS, %	−0.011	0.008	0.989 (0.974, 1.004)	−1.467	0.14
CI, L/min	0.021	0.032	1.021 (0.960, 1.087)	0.672	0.50
LVEDV, mL	0.000	0.002	1.000 (0.997, 1.003)	0.248	0.80
SV, mL	0.000	0.003	1.000 (0.994, 1.005)	−0.114	0.91

Abbreviations: AVF, Arteriovenous Fistula; AVG, Arteriovenous Graft; b, Regression Coefficient; CI, Cardiac Index; HR, Hazard Ratio; IVST, Interventricular Septal Thickness; LAD, Left Atrial Diameter; LVEDD, Left Ventricular End‐Diastolic Diameter; LVEDV, Left Ventricular End‐Diastolic Volume; LVEF, Left Ventricular Ejection Fraction; LVESD, Left Ventricular End‐Systolic Diameter; LVFS, Left Ventricular Fractional Shortening; LVPWT, Left Ventricular Posterior Wall Thickness; MPAD, Main Pulmonary Artery Diameter; PH, Pulmonary Hypertension; Ref, Reference Group; RVD, Right Ventricular Diameter; RVOT, Right Ventricular Outflow Tract; SE, Standard Error; SV, Stroke Volume; 95% CI, 95% Confidence Interval.

The Fine‐Gray model was constructed for multivariate analysis. Seven variables with *p* < 0.1 in the univariate analysis were included in the model, including PH probability stratification, age, LAD, LVESD, RVD, MPAD, and LVEF. Multivariate analysis results demonstrated that intermediate probability of PH (HR = 1.784, 95% CI 1.289–2.469, *p* < 0.001), high probability of PH (HR = 2.470, 95% CI 1.673–3.647, *p* < 0.001), age (HR = 1.024, 95% CI 1.012–1.037, *p* < 0.001), and RVD (HR = 1.053, 95% CI 1.003–1.105, *p* = 0.038) were independent risk factors for all‐cause mortality in AVF/AVG‐treated MHD patients (Table 10).

**Table 10:** Multivariate competing risk model for patients with AVF/AVG (Access = 1, *n* = 495).

Variables	Regression coefficient (b)	Standard Error (SE)	HR (95% CI)	z value	*p* value
Echocardiographic probability of PH grouping					
0: Low probability	Ref	—	—	—	—
1: Intermediate probability	0.579	0.166	1.784 (1.289, 2.469)	3.492	< 0.001**
2: High probability	0.904	0.199	2.470 (1.673, 3.647)	4.548	< 0.001**
Age, years	0.024	0.006	1.024 (1.012, 1.037)	3.880	< 0.001**
LAD, mm	0.010	0.012	1.010 (0.985, 1.035)	0.764	0.44
LVPWT, mm	0.004	0.018	1.004 (0.970, 1.040)	0.221	0.83
RVD, mm	0.051	0.025	1.053 (1.003, 1.105)	2.074	0.038*
MPAD, mm	0.005	0.024	1.005 (0.959, 1.053)	0.205	0.84
LVEF, %	−0.222	1.149	0.801 (0.084, 7.605)	−0.194	0.85

Abbreviations: AVF, Arteriovenous Fistula; AVG, Arteriovenous Graft; b, Regression Coefficient; HR, Hazard Ratio; LAD, Left Atrial Diameter; LVEF, Left Ventricular Ejection Fraction; LVPWT, Left Ventricular Posterior Wall Thickness; MPAD, Main Pulmonary Artery Diameter; PH, Pulmonary Hypertension; Ref, Reference Group; RVD, Right Ventricular Diameter; SE, Standard Error; 95% CI, 95% Confidence Interval.

### 3.7 Interaction Analysis Between PH Probability and Vascular Access Type

To evaluate whether the effect of PH on all‐cause mortality differed by vascular access type, we added multiplicative interaction terms (PH probability group × vascular access type) to the fully adjusted Fine‐Gray model. As shown in Table 11, the interaction term for intermediate PH probability × TCC (vs. AVF/AVG) was not statistically significant (HR = 0.87, 95% CI 0.52–1.45, *p* = 0.59). Similarly, the interaction term for high PH probability × TCC was not significant (HR = 0.71, 95% CI 0.36–1.39, *p* = 0.32). A likelihood ratio test comparing the model with both interaction terms to the model without them yielded *p* = 0.51. These findings indicate that the association between PH severity and mortality does not significantly differ between patients with TCC and those with AVF/AVG access.

**Table 11:** Interaction analysis between PH probability and vascular access type in the Fine‐Gray competing risk model (*n* = 749).

Variable	HR (95% CI)	*p* value
**Main effects**		
Intermediate PH probability (vs. low)	1.85 (1.40–2.45)	< 0.001
High PH probability (vs. low)	2.91 (2.05–4.13)	< 0.001
TCC (vs. AVF/AVG)	1.09 (0.82–1.45)	0.54
**Interaction terms**		
Intermediate PH × TCC	0.87 (0.52–1.45)	0.59
High PH × TCC	0.71 (0.36–1.39)	0.32
**Overall interaction *p*‐value (likelihood ratio test)**		**0.51**

*Note:* Model adjusted for age, LAD, RVD, sex, cause of kidney failure, systolic blood pressure, serum albumin, hemoglobin, phosphate, and CRP.

Abbreviations: AVF, Arteriovenous Fistula; AVG, Arteriovenous Graft; PH, Pulmonary Hypertension; TCC, Tunneled Cuffed Catheter.

## 4 Discussion

CKD patients on MHD face an extremely high risk of cardiovascular mortality, with echocardiographic probability of PH and vascular access type recognized as key modifiers of long‐term prognosis [17, 18]. However, prior survival analyses in MHD populations have rarely accounted for renal transplantation as a competing event, which may lead to biased effect estimates of risk factors. Furthermore, the heterogeneity in the association between echocardiographic probability of PH, cardiac structural remodeling, and mortality across different vascular access types remains poorly characterized. In this retrospective single‐center cohort of 749 MHD patients, we used a Fine‐Gray competing risk model (with renal transplantation as the competing event) to investigate the predictors of all‐cause mortality. Our key findings are threefold: First, echocardiographic probability of PH severity, age, LAD, and RVD were independent risk factors for all‐cause mortality in the overall cohort, with a stepwise reduction in long‐term survival across increasing echocardiographic probability of PH strata. Second, there was no significant difference in overall long‐term survival between patients with TCC and those with AVF/AVG. Third, the independent predictors of mortality differed significantly by vascular access: age and high echocardiographic probability of PH were the only independent risk factors in TCC‐treated patients, while age, intermediate/high echocardiographic probability of PH, and RVD were independent mortality predictors in the AVF/AVG group.

Traditional survival analyses, such as the Cox proportional hazards model, treat competing events (e.g., renal transplantation in this cohort) as censored observations, which can overestimate the effect size of risk factors for the primary endpoint of all‐cause mortality [19]. In MHD populations, renal transplantation fundamentally alters the disease trajectory and precludes the observation of dialysis‐related mortality, making it a nonnegligible competing event [20]. The Fine‐Gray competing risk model used in this study explicitly accounts for this competing event, providing a more unbiased and accurate estimation of the true association between potential predictors and all‐cause mortality in MHD patients. The overall 1‐year, 3‐year, 5‐year, and 10‐year all‐cause survival rates in our cohort were 82.22%, 67.71%, 60.60%, and 42.22%, respectively. Our 5‐year survival rate is consistent with the national data from the Chinese National Renal Data System (CNRDS) [21], while the 1‐year and 3‐year rates are slightly lower, which may be attributed to differences in study period, baseline comorbidity burden, and regional dialysis care standards. Globally, survival outcomes of MHD patients vary widely across economic regions, reflecting disparities in healthcare resources and dialysis quality [22]. Notably, we observed a strong gradient association between echocardiographic probability of PH severity and survival: the 10‐year survival rate was only 12.54% in the high echocardiographic probability of PH group, compared with 52.58% in the low echocardiographic probability of PH group, establishing echocardiographic probability of PH as a robust negative predictor of long‐term survival in MHD patients.

Age was the only shared independent risk factor for all‐cause mortality in both TCC and AVF/AVG groups, consistent with extensive prior literature [23, 24, 25]. Elderly MHD patients have a higher burden of comorbidities, poorer nutritional status, and elevated risk of dialysis‐related complications, all of which contribute to increased mortality. Age is also an established independent predictor of echocardiographic probability of PH development, further amplifying the mortality risk in this population [26]. Additionally, the lower likelihood of renal transplantation in elderly patients makes mortality a more prominent competing event, further justifying the use of the competing risk model in our analysis.

Clinical practice guidelines universally recommend AVF as the first‐line vascular access for MHD patients, with multiple early studies linking central venous catheter use to higher mortality risk [27, 28, 29]. However, our study found no significant difference in long‐term all‐cause survival between the TCC and AVF/AVG groups, aligning with findings from several recent contemporary cohorts [30, 31]. We postulate that this “survival paradox” arises from the fundamentally distinct risk profiles associated with each access type: TCC use is primarily linked to infection‐related mortality, while the persistent hemodynamic shunt from AVF/AVG drives adverse cardiac remodeling, echocardiographic probability of PH progression, and subsequent cardiogenic mortality. Our study suggests potential qualitative differences in the drivers of mortality across vascular access types, which may help explain the absence of an overall survival difference between the two groups. However, these observations are based on exploratory subgroup analyses rather than formal interaction testing, and should be considered hypothesis‐generating rather than definitive evidence of effect modification.

Echocardiographic probability of PH is highly prevalent across all stages of CKD, and previous studies, including the large Chronic Renal Insufficiency Cohort (CRIC) study, have consistently identified echocardiographic probability of PH as an independent risk factor for cardiovascular events and all‐cause mortality in CKD and dialysis patients [32, 33]. The key novel finding of our study is the significant modification effect of vascular access on the echocardiographic probability of PH‐mortality axis. In the TCC group, only high echocardiographic probability of PH was independently associated with mortality, suggesting that echocardiographic probability of PH in TCC‐treated patients is predominantly driven by baseline ESKD‐related factors, such as chronic volume overload and left heart disease. In contrast, both intermediate and high echocardiographic probability of PH, as well as RVD (a marker of right ventricular remodeling), were independent mortality predictors in the AVF/AVG group. This indicates that the continuous hemodynamic changes induced by AVF/AVG drive the parallel progression of echocardiographic probability of PH and right ventricular remodeling, which synergistically increase cardiogenic mortality risk. Prior research has focused on the overall association between echocardiographic probability of PH and mortality in MHD patients [34, 35], but has not characterized the modifying effect of vascular access on the echocardiographic probability of PH‐right heart‐mortality pathway, which represents a key advance of our work.

Several limitations of this study should be acknowledged. Our multivariable adjustment strategy has important limitations. Although we controlled for key demographic and echocardiographic variables, we were unable to adjust for several well‐established mortality predictors in MHD patients, including dialysis adequacy (single‐pool Kt/V, urea reduction ratio), residual renal function, normalized protein catabolic rate, and specific cardiovascular medications (renin‐angiotensin system inhibitors, beta‐blockers, statins), because these data were not systematically recorded in our retrospective database. Furthermore, forcing all clinically relevant covariates into the Fine‐Gray models would have resulted in an unacceptably low events‐per‐variable ratio (< 5 in subgroup analyses), risking model overfitting and unstable estimates. Consequently, our findings should be interpreted as independent prognostic associations rather than definitive causal effects, and the reported hazard ratios may reflect residual confounding from unmeasured or partially measured clinical factors. Prospective studies with standardized, comprehensive data collection are needed to confirm these relationships. In addition, the retrospective nature of this study precluded uniform documentation of all guideline‐recommended additional echocardiographic signs (e.g., TAPSE, IVC diameter, and septal flattening), and our PH probability stratification relied primarily on structural parameters (RVD, RVOT, MPAD, LAD) that were consistently available in historical TTE reports. Finally, subgroup analyses and effect modification. We performed separate competing risk models within TCC and AVF/AVG subgroups to explore potential differences in mortality predictors. However, we did not include a formal interaction term between PH probability and vascular access type in the overall model. This decision was based on the small number of patients with high PH probability in the TCC subgroup (*n* = 9, representing only 3.5% of the TCC cohort; see Table 1), which would result in severe data sparsity in the cross‐classified cells and preclude stable estimation of an interaction effect. Consequently, the observed differences between subgroups should be interpreted as exploratory and hypothesis‐generating, not as statistically confirmed effect modification.

## 5 Conclusion

In conclusion, using a competing risk model accounting for renal transplantation, we demonstrate that echocardiographic probability of PH severity, age, and markers of cardiac structural remodeling are independent risk factors for all‐cause mortality in MHD patients. Notably, while overall long‐term survival does not differ significantly between TCC and AVF/AVG groups, the drivers of mortality are highly vascular access–specific: mortality in TCC patients is predominantly predicted by baseline severe echocardiographic probability of PH, whereas mortality in AVF/AVG patients is driven by the combined effects of echocardiographic probability of PH progression and right ventricular remodeling. Our findings suggest that cardiovascular risk profiles may differ by vascular access type and support the need for further prospective studies to validate whether PH and right ventricular remodeling have differential prognostic implications in TCC versus AVF/AVG patients. For patients with AVF/AVG, regular echocardiographic monitoring of echocardiographic probability of PH and right ventricular structure, along with early targeted intervention, may reduce cardiogenic mortality and improve long‐term outcomes.

## Author Contributions

All authors participated in the design, interpretation of the studies and analysis of the data and review of the manuscript. J Z drafted the work and revised it critically for important intellectual content; Z Y was responsible for the acquisition, analysis and interpretation of data for the work; J Z and D H made substantial contributions to the conception or design of the work. All authors read and approved the final manuscript.

## Funding

The authors have nothing to report.

## Ethics Statement

This study was approved by the Ethics Committee of the First Affiliated Hospital of Guangxi Medical University (Approval Number: 2026‐E0230). This study was performed in line with the principles of the Declaration of Helsinki. All methods were carried out in accordance with relevant guidelines and regulations.

## Consent

Informed consent was obtained from all individual participants included in the study.

## Conflicts of Interest

The authors declare no conflicts of interest.

## Supporting information


**Table S1:** Missing data for key clinical, laboratory, and echocardiographic variables (n = 749).

## Data Availability

The data that support the findings of this study are available from the corresponding author upon reasonable request.
