# Supplementary material for: Echocardiographic Probability of Pulmonary Hypertension, Cardiac Structural Alterations and All‐Cause Mortality in Maintenance Hemodialysis Patients: A Single‐Center Retrospective Cohort Study With Competing Risk Analysis
Source: Clin Cardiol. 2026 Jul 18;49(7):e70415. doi: 10.1002/clc.70415 (PMC13379943; doi:10.1002/clc.70415)
Supplement: Supplementary file 1 — Table S1: Missing data for key clinical, laboratory, and echocardiographic variables (n = 749). [file CLC-49-e70415-s001.docx]

Supplementary Table 1. Missing data for key clinical, laboratory, and echocardiographic variables (n=749)

| **Variable** | **Number of patients with available data (n)** | **Number of missing values (n)** | **Proportion of missing (%)** |
| --- | --- | --- | --- |
| Demographic and clinical |  |  |  |
| Age | 749 | 0 | 0.0 |
| Sex | 749 | 0 | 0.0 |
| Smoking history | 749 | 0 | 0.0 |
| Treatment duration | 749 | 0 | 0.0 |
| Cause of kidney failure | 749 | 0 | 0.0 |
| Vascular access type | 749 | 0 | 0.0 |
| Systolic blood pressure | 718 | 31 | 4.1 |
| Laboratory parameters |  |  |  |
| Serum albumin | 706 | 43 | 5.7 |
| Hemoglobin | 711 | 38 | 5.1 |
| Serum phosphate | 697 | 52 | 6.9 |
| C-reactive protein (CRP) | 665 | 84 | 11.2 |
| Serum creatinine | 740 | 9 | 1.2 |
| Blood urea nitrogen | 738 | 11 | 1.5 |
| Echocardiographic parameters |  |  |  |
| LAD | 749 | 0 | 0.0 |
| LVEDD | 749 | 0 | 0.0 |
| LVESD | 749 | 0 | 0.0 |
| RVD | 749 | 0 | 0.0 |
| MPAD | 749 | 0 | 0.0 |
| LVEF | 749 | 0 | 0.0 |
| PH probability group | 749 | 0 | 0.0 |

Note: LAD: Left Atrial Diameter; LVEDD: Left Ventricular End-Diastolic Diameter; LVESD: Left Ventricular End-Systolic Diameter; RVD: Right Ventricular Diameter; MPAD: Main Pulmonary Artery Diameter; LVEF: Left Ventricular Ejection Fraction; PH: Pulmonary Hypertension.
